# Inflammatory, Nutritional, and Atherogenic Profiles Associated with Histologic Activity in Inflammatory Bowel Disease

**DOI:** 10.3390/biomedicines14040740

**Published:** 2026-03-24

**Authors:** Dilek Ayvaz, Muammer Bilici

**Affiliations:** 1 Department of Gastroenterology, Faculty of Medicine, Zonguldak Bulent Ecevit University, Zonguldak 67100, Turkey; drdilekmalkoc@gmail.com; 2 Department of Internal Medicine, Faculty of Medicine, Zonguldak Bulent Ecevit University, Zonguldak 67100, Turkey

**Keywords:** inflammatory bowel disease, histologic activity, nutrition, inflammation, systemic inflammation response index, atherogenic index of plasma, lipids

## Abstract

**Background/Objectives**: Histologic remission has emerged as a key treatment target in inflammatory bowel disease (IBD), but routine assessment requires repeated endoscopy and biopsies. Blood-based indices reflecting inflammation, nutritional status and atherogenic risk are inexpensive and widely available, yet their integrated contribution to histologic activity remains unclear. This study addresses this gap by simultaneously analyzing a broad panel of 44 variables—including nutritional status indicators, CBC-derived inflammation indices, and atherogenic lipid indices—in IBD patients. **Methods**: In this retrospective study, 100 patients with IBD (50 Crohn’s disease [CD], 50 ulcerative colitis [UC]) without additional comorbidities and with concomitant histologic assessment were analyzed. Histologic activity was coded as active vs. remission. At the time of biopsy, the complete blood count, biochemistry and lipid profile were used to calculate immuno-nutritional indices (CONUT score, prognostic nutritional index), inflammatory indices (neutrophil-to-platelet ratio, platelet-to-lymphocyte ratio, lymphocyte-to-monocyte ratio [LMR], systemic immune-inflammation index, systemic immune-inflammation index, systemic inflammation response index [SIRI], aggregate index of systemic inflammation, C-reactive protein to albumin ratio) and atherogenic indices (atherogenic index of plasma [AIP], triglyceride-to-HDL cholesterol ratio). Variable selection was performed separately for CD and UC using least absolute shrinkage and selection operator (LASSO) regression and sparse partial least squares discriminant analysis (sPLS-DA). Independently associated predictors were then entered into multivariable logistic regression models, and their discriminative performance was evaluated using ROC analysis with bootstrap-derived 95% confidence intervals. **Results**: LASSO analysis identified a broadly similar systemic profile associated with histologic activity in CD and UC, dominated by the CONUT score, SIRI, AIP, LMR and red blood cell parameters, whereas demographic features and most routine biochemical markers were shrunk towards zero. Cross-validated AUCs for the LASSO models were 0.93 in CD and 0.87 in UC. sPLS-DA confirmed this pattern: CONUT, SIRI and AIP consistently showed the highest variable importance in projection scores and loadings on the first latent component. In multivariable regression, the CONUT score, SIRI and AIP remained independent predictors of histologic activity in CD, while hematocrit, CONUT score, SIRI and AIP were independently associated with histologic activity in UC. In ROC analysis, AUCs for CONUT, SIRI and AIP were 0.81, 0.89 and 0.87 in UC, and 0.72, 0.82 and 0.83 in CD, respectively. **Conclusions**: Histologic activity in IBD is characterized by a coupled systemic profile in which immuno-nutritional compromise (captured by CONUT) forms the core signal, supplemented by systemic inflammation (SIRI) and atherogenic dyslipidemia (AIP). These readily available blood-based indices may help to approximate histologic disease activity in clinical practice. However, considering that comorbid diseases may affect these indices, the strict exclusion criteria applied in this study may limit the generalizability of the findings among patients with IBD. Consequently, further validation in larger prospective cohorts is warranted.

## 1. Introduction

Inflammatory bowel disease (IBD) is a chronic, relapsing-remitting inflammatory disorder of the gastrointestinal tract [1]. It encompasses two main subtypes: ulcerative colitis (UC) and Crohn’s disease (CD). UC causes continuous inflammation confined to the colon (large intestine), primarily affecting the superficial mucosal layer, whereas CD is characterized by transmural inflammation that can involve any part of the GI tract from the mouth to the anus, often in a patchy distribution with “skip” lesions [2,3]. The precise etiology of IBD remains unclear, but it is thought to arise from a complex interplay of genetic predisposition, environmental triggers, gut microbiota dysbiosis, and aberrant immune responses [4]. Disease activity and severity are typically evaluated using clinical indices that integrate patient symptoms, laboratory inflammatory markers, and endoscopic findings [5].

IBD’s chronic inflammation has far-reaching systemic effects. Malnutrition is a well-recognized complication of IBD, resulting from reduced oral intake, malabsorption, and the catabolic effects of prolonged inflammation [6]. Indeed, studies report that roughly 25–70% of patients with active IBD show evidence of malnutrition, a condition associated with worse clinical outcomes and the need for nutritional support [7]. The persistent inflammatory state can also disrupt metabolic processes, including lipid metabolism. Paradoxically, active IBD is often associated with lower levels of total cholesterol, LDL, and HDL cholesterol compared to healthy controls [8]. These depressed lipid levels are thought to reflect malnourishment and inflammatory consumption of lipids [9]. However, the persistent inflammatory state may render the lipid profile more atherogenic in effect—IBD patients have an elevated risk of atherosclerotic cardiovascular disease, largely attributed to chronic systemic inflammation and immune activation [10].

Numerous studies have explored inflammatory and nutritional indices as markers of IBD activity. Recent meta-analyses show that complete blood count-derived inflammatory indices such as neutrophil-to-platelet ratio (NLR), platelet-to-lymphocyte ratio (PLR), or composite inflammatory indices like the C-reactive protein to albumin ratio (CAR) are significantly elevated in patients with active IBD and correlate with disease severity [11]. Such indices are inexpensive and readily available, making them attractive adjuncts for disease monitoring. Likewise, nutritional status indices have prognostic value in IBD: tools like the prognostic nutritional index (PNI) and controlling nutritional status (CONUT) score consistently identify malnutrition as an independent risk factor for IBD flare-ups [12]. In contrast, atherogenic lipid indices (such as ratios of cholesterol fractions or the atherogenic index of plasma) have been less extensively studied in IBD, even though dyslipidemia and cardiovascular risks are known concerns in this patient population [13]. Taken together, these findings suggest that active IBD is linked to poor nutritional status and higher systemic inflammatory indices and atherogenic lipid profiles, indicative of an amplified pro-inflammatory milieu.

However, most previous studies have examined inflammatory, nutritional, and lipid-related markers in isolation, and their combined relationship with histologic activity in IBD remains insufficiently defined. This represents an important clinical gap, because histologic activity has emerged as a meaningful therapeutic target and prognostic indicator, yet its assessment still depends on repeated endoscopy and biopsy. Given that intestinal inflammation, malnutrition, and lipid disturbances are biologically interconnected manifestations of active IBD, an integrated evaluation of these domains may better capture ongoing microscopic disease activity than any single marker alone. Therefore, this study addresses this gap by simultaneously analyzing a broad panel of 44 variables—including nutritional status indicators, CBC-derived inflammation indices, and atherogenic lipid indices—in IBD patients. By investigating this broad panel of parameters, we aim to better characterize the multifaceted nature of IBD in active disease and identify robust predictors of disease activity. This integrative approach highlights the importance of viewing IBD not only as a localized gut inflammation but as a systemic condition with nutritional and metabolic dimensions.

## 2. Materials and Methods

This retrospective study included adult patients with a diagnosis of IBD who were followed at the IBD outpatient clinic of Gastroenterology Department between 1 January 2022 and 31 December 2024. The study protocol complied with the principles of the Declaration of Helsinki and was approved by the Bulent Ecevit University Hospital Clinical Research Ethics Committee (approval date: 17 December 2025, decision no: 2025/22). Owing to the retrospective design, the Ethics Committee waived the requirement for obtaining informed consent.

### 2.1. Study Design and Population

Over the study period, 186 individuals with a confirmed diagnosis of IBD were retrospectively screened. Eligible participants were adults aged 18–90 years with IBD, regardless of sex. Patients were excluded if they had evidence of active infection (including intestinal parasitic infestations or other infectious foci), chronic inflammatory disorders (e.g., rheumatologic or chronic infectious diseases), cardiovascular or cerebrovascular disease, hepatic or renal failure, thyroid dysfunction, pregnancy, a history of bowel surgery, use of systemic immunosuppressive agents for non-IBD indications (such as rheumatologic or oncologic diseases), or incomplete/missing clinical/laboratory data. These exclusion criteria were applied to reduce major non-IBD determinants of the inflammatory, nutritional, and atherogenic indices evaluated in this study. In particular, active infection, chronic inflammatory comorbidities, non-IBD immunosuppressive therapy, hepatic or renal failure, thyroid dysfunction, established cardiovascular/cerebrovascular disease, pregnancy, and prior bowel surgery could independently influence blood counts, albumin, lipid parameters, nutrient absorption, or disease phenotype [14,15,16,17,18,19]. Accordingly, the final cohort was intended to provide a more internally consistent assessment of the association between histologic activity and the studied composite markers. Following application of these criteria, 100 patients were included in the final analysis, comprising age- and sex-matched groups of 50 patients with UC and 50 patients with CD.

### 2.2. Data Collection and Definitions

Demographic and clinical characteristics were obtained from the hospital’s electronic medical records and archived patient files. The collected datasets are presented in detail in Supplementary Table S1. Briefly, a total of 44 variables were analyzed, including age, gender, body mass index (BMI), smoking history, clinical activity score, IBD localization, laboratory parameters, and composite indices derived from these parameters, such as nutritional, inflammatory, and atherogenic lipid indices. To assess disease activity, a detailed clinical history obtained at admission was reviewed. To minimize treatment-related confounding, only baseline laboratory data obtained at the initial evaluation were analyzed. These fasting blood samples were drawn on the same day as endoscopic biopsy, before initiation of IBD. Nutritional status was classified according to the CONUT score as normal (0–1), mild malnutrition (2–4), moderate malnutrition (5–8), and severe malnutrition (9–12) [20].

Endoscopic examinations were performed by a single experienced endoscopist using a FUJINON XL-4450 colonoscope (Fujifilm Medical Co., Tokyo, Japan). Disease severity was graded with the Harvey–Bradshaw Index (HBI) in patients with CD and with the Mayo scoring system (Disease Activity Index, DAI) in patients with UC [21,22]. Histologic data were obtained retrospectively from finalized colorectal mucosal biopsy reports documented in the electronic medical records and archived patient files. Because this study was based on retrospective chart review, the exact number and segmental distribution of biopsy samples, including the number obtained from the most inflamed area, were not consistently documented and therefore could not be standardized across all patients. Histologic evaluations were performed in routine clinical practice by a single pathologist. In patients with UC, histologic activity was assessed using the Nancy Index as recorded in the pathology reports. In patients with CD, histologic status was classified dichotomously as active disease or remission. Histologic activity was defined by the presence of neutrophil infiltration, cryptitis, crypt abscesses, erosions, or ulceration, whereas histologic remission was defined as the absence of these features, with at most chronic inflammatory or architectural changes.

### 2.3. Composite Inflammatory, Nutritional, and Atherogenic Indices

Composite indices reflecting systemic inflammation, nutritional status, and atherogenic lipid profile were calculated using routinely measured hematological and biochemical parameters. The formulas for NLR, PLR, lymphocyte-to-monocyte ratio (LMR), systemic immune-inflammation index (SII), systemic inflammation response index (SIRI), aggregate index of systemic inflammation (AISI), CAR, PNI, CONUT score, atherogenic index of plasma (AIP), and triglyceride-to-HDL cholesterol ratio (TG/HDL) are summarized in Supplementary Table S2.

### 2.4. Statistical Analysis

All statistical procedures were conducted using SPSS version 24.0 (IBM Corp., Armonk, NY, USA) and R version 4.3.0 (R Foundation for Statistical Computing, Vienna, Austria). The distribution of continuous variables was examined with the Kolmogorov–Smirnov test. Continuous data are reported as mean ± standard deviation (SD) when normally distributed, and as median with interquartile range (IQR) when the distribution was non-normal; categorical data are presented as counts and percentages. Comparisons between groups were made with Student’s *t*-test for normally distributed variables and the Mann–Whitney U test for skewed variables, whereas categorical variables were analyzed using the Chi-square test or Fisher’s exact test, as appropriate.

Variable reduction was carried out using two complementary approaches: least absolute shrinkage and selection operator (LASSO)-penalized regression and sparse partial least squares discriminant analysis (sPLS-DA) [23,24]. In the LASSO models, a logistic regression with an L1 penalty was fitted using the glmnet package in R. The regularization parameter [log10(C)] was tuned by 5-fold stratified cross-validation using the area under the receiver operating characteristic curve (AUC-ROC) as the optimization criterion (cv.glmnet() function, type.measure = “auc”). All continuous predictor variables were standardized (z-transformed) before modeling. Trajectories of standardized regression coefficients were plotted across the range of log10(C) values, and predictors with non-zero coefficients at the selected penalty level were carried forward to subsequent multivariable analyses [24]. In parallel, sPLS-DA was conducted using the mixOmics package in R. The splsda() function was used to derive latent components that best discriminated histologically active from inactive disease. The optimal number of latent dimensions and features per component was determined by leave-one-out cross-validation (tune.splsda()), and for each model, variable importance in projection (VIP) scores, percentage of explained variance, and classification accuracy were obtained [25,26]. Variables with VIP scores ≥ 1 were considered relevant contributors to class separation [26,27]. Discriminative performance of selected predictors was further evaluated using receiver operating characteristic (ROC) analysis, with AUC values and corresponding 95% confidence intervals (CIs) estimated via nonparametric bootstrap resampling (1000 iterations) [28,29].

The variables retained after variable selection were entered into multivariable logistic regression models to determine independent predictors of histologically active disease. Results were expressed as adjusted odds ratios (ORs) with corresponding 95% CIs. ROC curve analyses were then used to assess the discriminative performance of individual laboratory parameters and composite indices for the presence (and, in secondary analyses, severity) of histologic activity in IBD. Optimal cutoff values were derived using the Youden index, and differences between AUCs of independent predictors were compared with the DeLong test [30].

A two-tailed *p*-value < 0.05 was considered statistically significant.

## 3. Results

### 3.1. Patient Characteristics

Among 100 IBD patients (CD n = 50; UC n = 50), the overall mean age was 47.3 ± 13.7 years, BMI 26.1 ± 5.0 kg/m^2^, smoking 23.0%, and the median disease activity score was 3.0. Histologic remission was present in 40.0% overall. In CD, disease location was ileal in 44.0%, colonic in 38.0%, and ileocolonic in 18.0%; in UC, involvement was proctitis in 48.0%, left-sided in 30.0%, and extensive/pancolitis in 22.0%. Demographic and clinical characteristics were comparable between the CD and UC groups (Table 1).

### 3.2. Parameters Associated with Histologic Activity

CD patients with active histology showed higher leukocytes (8.9 ± 2.8 vs. 6.9 ± 1.9, *p* = 0.008) and monocytes (0.7 ± 0.3 vs. 0.5 ± 0.1, *p* = 0.003), and had lower hemoglobin (12.2 ± 1.9 vs. 13.4 ± 1.4, *p* = 0.023) and hematocrit (37.0 ± 5.5 vs. 40.4 ± 3.5, *p* = 0.011), compared with those in histologic remission. Inflammation markers were consistently worse in the active group: neutrophils (5.7 vs. 4.5, *p* < 0.001), ESR (33.0 vs. 11.0, *p* = 0.003), and CRP (19.9 vs. 6.3, *p* = 0.007). Nutritional/lipid profiles also deteriorated: albumin (2.9 ± 0.8 vs. 4.3 ± 0.3, *p* < 0.001) and HDL (39.0 vs. 53.0, *p* = 0.004) were lower, whereas triglycerides were higher (median 123.0 vs. 82.0, *p* = 0.018). Liver enzymes were modestly lower in the active group (ALT 12.0 vs. 15.0, *p* = 0.031; AST 14.0 vs. 17.0, *p* = 0.047). Composite inflammatory indices were markedly unfavorable with activity: NLR (4.0 vs. 1.8, *p* < 0.001), LMR (2.7 vs. 4.5, *p* < 0.001), SII (1104.9 vs. 579.0, *p* < 0.001), SIRI (1.8 vs. 0.9, *p* < 0.001), AISI (688.8 vs. 293.4, *p* < 0.001), and CAR (3.0 vs. 1.5, *p* = 0.025). Nutritional indices also reflected worse status: CONUT (2.0 vs. 1.0, *p* = 0.006) was higher and PNI lower (47.9 ± 10.3 vs. 54.0 ± 7.1, *p* = 0.024). Atherogenicity increased with activity: AIP (median 0.5 vs. 0.2, *p* < 0.001) and TG/HDL ratio (3.0 vs. 1.5, *p* < 0.001) were higher (Supplementary Table S3).

Compared with patients in histologic remission, those with active histology showed higher leukocytes (8.2 vs. 6.1, *p* = 0.010), neutrophils (5.3 vs. 4.1, *p* = 0.007) and monocytes (0.7 vs. 0.4, *p* = 0.002). Albumin was lower (3.4 ± 1.0 vs. 4.4 ± 0.2 g/dL, *p* < 0.001), while triglycerides were higher (146.0 vs. 68.0 mg/dL, *p* < 0.001). Composite inflammatory indices were consistently worse with activity: LMR was lower (2.9 ± 1.5 vs. 4.5 ± 2.1, *p* = 0.003), whereas SII (834.0 vs. 668.0, *p* = 0.040), SIRI (2.3 ± 1.1 vs. 0.9 ± 0.5, *p* < 0.001), AISI (581.0 vs. 202.5, *p* < 0.001) and CAR (4.1 vs. 1.1, *p* = 0.026) were higher. Nutritional status was poorer with activity: CONUT score was higher (3.0 vs. 2.0, *p* < 0.001) and PNI lower (49.2 ± 7.8 vs. 53.6 ± 4.8, *p* = 0.034). Atherogenicity increased: AIP (0.5 vs. 0.2, *p* < 0.001) and TG/HDL ratio (3.4 vs. 1.4, *p* < 0.001) were higher (Supplementary Table S4).

### 3.3. Parameters Selection for Histologic Activity

**Figure 1:**
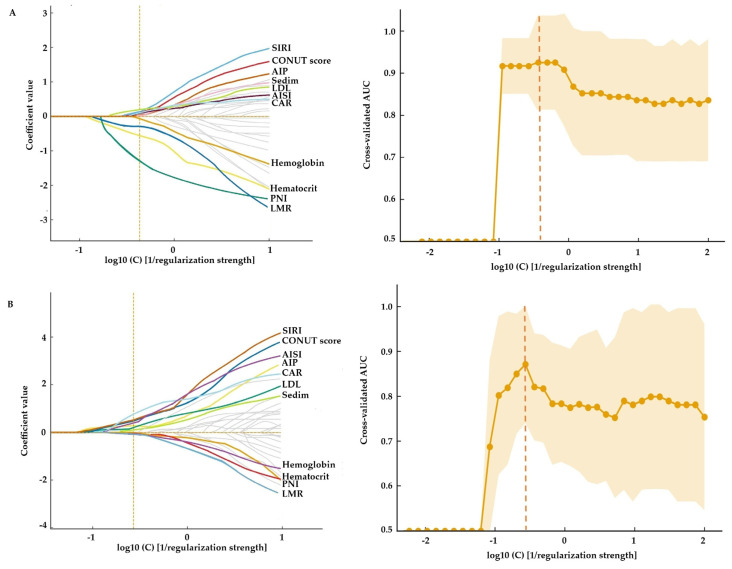
LASSO-based selection of parameters associated with histologically active disease in inflammatory bowel disease. (**A**) Crohn’s disease (CD) and (**B**) ulcerative colitis (UC). Left panels show L1-penalized logistic regression coefficient paths as a function of log10(C) 1/regularization strength; colored lines represent variables with non-zero coefficients at the selected penalty level, whereas gray lines denote variables that are shrunk towards zero. Right panels display the 5-fold stratified cross-validated area under the ROC curve (AUC) across log10(C) values, with solid lines indicating mean AUC and shaded bands denoting ±1 SD. The vertical dashed lines mark the selected penalty levels: log10(C) = −0.37 for the CD model (AUC 0.93) and log10(C) = −0.57 for the UC model (AUC 0.87). At these penalty levels, only variables with non-zero coefficients were regarded as informative predictors and were carried forward into subsequent multivariable modeling.

To identify the key parameters associated with histologic activity and to reduce model dimensionality, we first applied LASSO-penalized logistic regression separately in UC and CD cohorts. This approach allowed us to shrink irrelevant coefficients toward zero and retain only the most informative demographic, clinical, and laboratory variables. LASSO regression identified a largely overlapping risk profile for histologically active disease in CD (Figure 1A; optimal log10(C) = −0.37) and UC (Figure 1B; optimal log10(C) = −0.57). Of the 44 candidate variables, only a limited set retained non-zero coefficients at the optimal penalty. These comprised nutritional indices (CONUT score, PNI), inflammatory markers (sedim, LMR, SIRI, AISI, CAR), lipid-related measures (AIP and LDL), and red blood cell parameters (hemoglobin and hematocrit). In contrast, demographic characteristics and most routine biochemical parameters were shrunk towards zero and eliminated from the models. The LASSO-based models showed good discrimination for histologically active disease, with an AUC of 0.87 (95% CI 0.76–0.99) in UC and 0.93 (95% CI 0.83–1.00) in CD (Figure 1).

**Figure 2:**
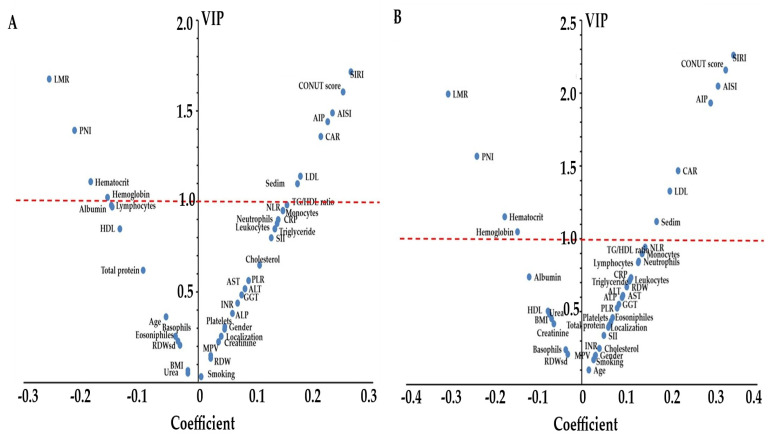
sPLS-DA V-plots of systemic inflammatory, nutritional, and atherogenic markers associated with histologic activity in inflammatory bowel disease. (**A**) Crohn’s disease and (**B**) ulcerative colitis. Each point represents one predictor variable, plotted according to its loading (coefficient) on the first sPLS-DA latent component (*x*-axis) and its variable importance in projection (VIP) score (*y*-axis). The horizontal red dashed line indicates the conventional VIP threshold of 1.0, above which variables are considered to have high discriminative importance. Abbreviations: see Table 1.

We also used sPLS-DA to visualize the multivariate separation between active and quiescent disease and to derive variable importance profiles for the selected markers. In both diseases, a single latent component provided adequate discrimination between histologic remission and active disease. For CD, this component explained 80% of the variance in predictor variables and 72% of the variance in the outcome, with a cross-validated AUC of 0.93 (95% CI 0.83–1.00) and 80% (95% CI 70–94%) of patients correctly classified. For UC, the corresponding values were 83% and 74%, with an AUC of 0.87 (95% CI 0.76–0.99) and 83% (95% CI 71–93%) correct classification. The latent component in both CD and UC was mainly driven by a consistent pattern of inflammatory, nutritional and atherogenic markers. Variables with VIP ≥ 1.0 that contributed positively to histologic activity included CONUT score, SIRI, AISI, AIP, CAR, LDL cholesterol and sedim, whereas LMR and PNI showed strong negative loadings, indicating inverse associations with active disease (Figure 2, Table 2).

Based on variables selected through both LASSO and PLS-DA analyses, multivariable logistic regression analysis revealed that SIRI (OR = 1.27, 95% CI = 1.07–1.49, *p* = 0.005), CONUT score (OR = 1.57, 95% CI = 1.03–2.42 *p* = 0.018), and AIP (OR = 1.05, 95% CI = 1.01–1.09, *p* = 0.023) levels independently predicted the histologic activity in CD, while hematocrit (OR = 0.77, 95% CI = 0.62–96, *p* = 0.022), SIRI (OR = 1.26, 95% CI = 1.06–1.50 *p* = 0.008), CONUT score (OR = 4.21, 95% CI = 1.47–12.05, *p* < 0.001), and AIP (OR = 1.09, 95% CI = 1.03–1.175, *p* = 0.006) were independent predictors of histologic activity in UC (Table 3).

**Figure 3:**
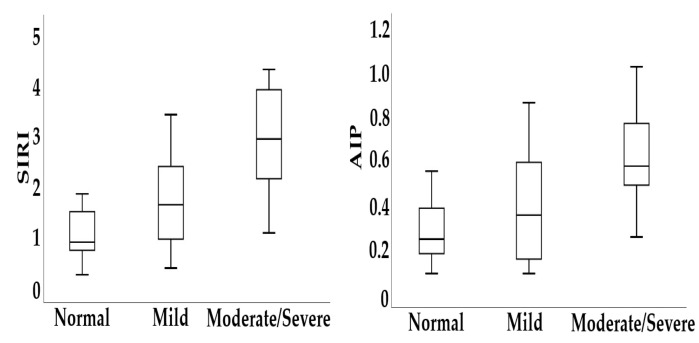
Distribution of SIRI and AIP levels according to nutritional status in patients with IBD.

**Figure 4:**
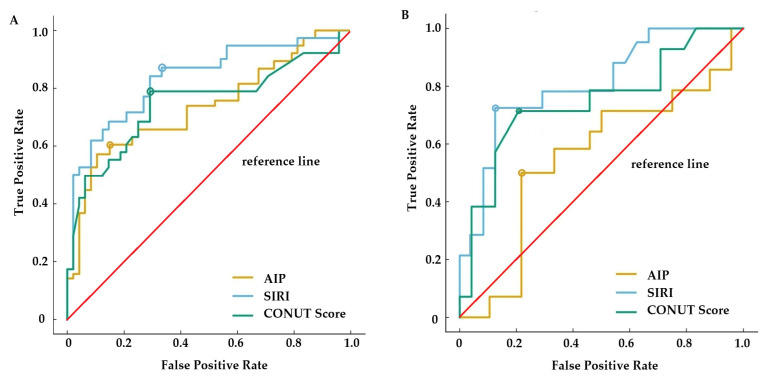
Diagnostic performance of CONUT score, SIRI, and AIP in predicting the histologically active disease in patients with CD (**A**) and UC (**B**). Colored circles indicate the optimal thresholds determined by maximizing Youden’s index for each ROC curve.

In IBD patients with moderate/severe malnutrition, both SIRI and AIP levels were higher compared with those with mild malnutrition and those with normal nutritional status (Figure 3 and Figure 4).

In both CD and UC patients, ROC analysis revealed that SIRI had the highest AUC value in predicting histologic disease activity (CD: SIRI_AUC_ = 0.86 vs. CONUT score_AUC_ = 0.78, *p* = 0.011; vs. AIP_AUC_ = 0.73, *p* < 0.001; UC: SIRI_AUC_ = 0.78 vs. CONUT score_AUC_ = 0.72, *p* = 0.034; vs. AIP_AUC_ = 0.60, *p* < 0.001). CONUT score showed superior diagnostic performance compared to AIP in predicting histologic disease activity (*p* = 0.018 for CD: *p* < 0.001 for UC) (Figure 4). Table 4 presents the optimal cutoff values, sensitivity, specificity, positive predictive value (PPV), negative predictive value (NPV), and AUC values for the three indices in patients with CD and UC.

## 4. Discussion

This study comprehensively characterizes the systemic profiles associated with histologic disease activity in IBD, emphasizing the interplay between inflammation, nutritional status, and atherogenic risk. By analyzing 44 routinely available laboratory markers across well-phenotyped cohorts of CD and UC, we identified a core set of variables—particularly the SIRI, the CONUT score, and the AIP—that were consistently elevated in histologically active patients. These markers not only reflected disease activity but also independently predicted histologic inflammation in multivariable models. Importantly, both SIRI and AIP increased with worsening nutritional status, underscoring the multidimensional burden of active disease. Our findings suggest that histologic activity in IBD may be captured through an integrated panel of systemic indices, offering a potentially valuable adjunct to endoscopic and clinical assessments.

Histological inflammation in IBD is increasingly recognized as a critical driver of disease outcomes. Persistent microscopic inflammation has been identified as a key factor behind relapses, steroid dependency, and complications, whereas achieving histologic remission is associated with better long-term outcomes [31]. Notably, up to 30% of patients in endoscopic remission still have ongoing histologic activity [32]. This underscores the importance of monitoring histologic disease activity in IBD and the need for reliable surrogate markers. Active IBD at the histological level is characterized by intense immune cell infiltration in the gut mucosa, particularly neutrophils, monocytes/macrophages, and lymphocytes, which drive tissue injury and inflammation [33]. Neutrophils, in particular, are a hallmark of acute inflammatory activity in the intestinal mucosa, and their presence (e.g., in crypt abscesses or epithelial layers) correlates with disease severity [34]. This localized inflammation is mirrored systemically: patients with active disease often exhibit elevated markers of systemic inflammation such as higher neutrophil and monocyte counts and a relative lymphopenia, which manifest in indices like an increased NLR or a decreased LMR [11,35]. Inflammatory cell-based indices have also been highlighted by prior studies. A 2021 meta-analysis showed that NLR levels were significantly higher in patients with IBD compared with healthy controls and increased with the severity of clinical disease activity [36]. Consistent with these findings, Feng et al. reported in a study from China that both NLR and PLR levels were higher in patients with active UC compared with those in remission [37]. Another meta-analysis published in 2025, including 35 studies, demonstrated that indices like NLR, PLR, and the CAR are elevated in active IBD and correlate with disease activity [11]. Cesaro et al. reported that CAR showed the highest accuracy for histologic activity, whereas SIRI and NLR had only modest discriminatory performance [38]. A study from Iraq involving newly diagnosed patients with CD reported that a low LMR was predictive of disease activity [39]. Similarly, a recent meta-analysis including 23 cohort studies with 3550 IBD patients confirmed that NLR and PLR levels were significantly elevated in IBD compared with healthy individuals and were higher during active disease than remission, while LMR showed the opposite pattern, being lower in active disease. The same analysis also reported a moderate diagnostic performance of these inflammatory indices for predicting IBD activity (pooled AUC ≈ 0.72), supporting their potential role as accessible biomarkers for disease monitoring [40]. Consistent with this evidence, our results demonstrated higher NLR and lower LMR values in patients with histologically active disease. Notably, these indices lost significance in multivariable regression analyses. This likely reflects the inclusion of more integrative inflammatory markers, such as SII and SIRI, which combine multiple leukocyte subtypes within a single metric. Because these composite indices simultaneously capture neutrophil-driven innate immune activation, monocyte-mediated inflammatory signaling, and lymphocyte suppression, they may better represent the complex systemic inflammatory milieu associated with histologically active IBD than simpler two-component ratios.

Increased SII and SIRI levels were associated with histologically active disease in both CD and UC patients. A study in patients with UC reported that SII levels were higher in active disease, with a sensitivity of 68.1% and specificity of 91.2%. However, SIRI was not assessed in that study [41]. Comparable findings have also been reported among patients with CD [42]. A case-control study demonstrated that SII correlated positively with endoscopic scores and predicted the endoscopic index of severity in UC patients, whereas NLR and PLR did not show these associations. Nevertheless, SIRI was not included among the evaluated indices in that study [43]. In a cohort comprising 210 IBD patients, higher SIRI levels were identified as an independent predictor of disease activity [44]. Another comprehensive study involving patients with IBD reported that SIRI showed better diagnostic performance than SII in predicting both endoscopic and histologic activity [38]. However, some studies have reported conflicting findings. In a study assessing comprehensive inflammatory indices for predicting disease activity in patients with UC, both SII and SIRI were found to be higher in patients with active disease, although SII showed better diagnostic performance than SIRI [45]. Carrillo-Palau et al. found that SIRI was not significantly related to disease activity in a cohort largely composed of patients with low-to-moderate outpatient disease [46]. In contrast, SIRI emerged as an independent marker of histologic activity in both UC and CD in our cohort. This discrepancy may reflect several methodological differences: our analysis was biopsy-paired, used histologic activity as the primary endpoint, and simultaneously evaluated nutritional and lipid-related domains rather than assessing inflammatory indices in isolation. The superior diagnostic performance of SIRI is consistent with prior evidence that elevated peripheral neutrophil/monocyte counts and reduced lymphocytes reflect active IBD. The tight association of neutrophil activation, monocytes, and monocyte-derived macrophages with IBD pathogenesis may underlie the potential utility of SIRI in this setting. Neutrophils infiltrate the intestinal mucosa and release proteases, reactive oxygen species, and neutrophil extracellular traps, directly damaging the epithelium and amplifying inflammation (often forming crypt abscesses), while circulating monocytes migrate into the gut and differentiate into pro-inflammatory macrophages that secrete cytokines (e.g., tumor necrosis factor-α, interleukin-6), sustaining the inflammatory milieu and contributing to tissue destruction [47]. SIRI reflects this immune imbalance by integrating high myeloid cell counts relative to suppressed lymphocytes [46]. Consistent with this shared inflammatory cascade, we also observed that UC and CD shared a broadly similar laboratory profile when histologically active, consistent with a common pathophysiologic response to inflammation. Notably, there are generally no major differences in nutritional or inflammatory parameters between active UC and CD patients [48]. This suggests a convergent pathway in which active mucosal inflammation—regardless of IBD subtype—triggers systemic inflammation and metabolic derangements to a comparable extent.

Beyond inflammatory cell-based indices, nutritional status represents another key dimension of disease activity in IBD. Among the 44 clinical and laboratory variables we analyzed, those reflecting systemic inflammation and nutritional/metabolic status emerged as most indicative of histologic activity. Patients with active CD and UC showed reduced PNI and elevated CONUT scores. This aligns with emerging evidence that malnutrition is both prevalent in IBD and correlated with disease severity. In fact, malnutrition is common during IBD flares, with one study reporting up to 25–70% of patients undernourished by various criteria [7]. Another study showed that UC patients had elevated CONUT scores, that over 90% were at risk of malnutrition, and that these individuals more often presented with moderate-to-severe disease activity [49]. In a study conducted in patients with active UC, PNI values were inversely correlated with disease severity [12]. On the other hand, chronic inflammation contributes to malnutrition through reduced oral intake, malabsorption, and hypermetabolic state. IBD patients frequently suffer protein–calorie undernutrition and micronutrient deficiencies, especially during active flares. Malnutrition can, in turn, impair mucosal immunity and wound healing, thereby exacerbating inflammation—creating a vicious cycle between nutritional status and disease activity [50]. This bidirectional interplay helps explain why systemic features like weight loss, hypoalbuminemia, and even metabolic changes accompany histologically active IBD. De-León-Rendón et al. reported that higher CONUT scores were associated with moderate-to-severe UC and with higher CRP levels, while Lian et al. showed that CONUT scores correlated with CRP and NLR, although its discrimination for clinical activity was only modest [49,51]. Our findings are consistent with these observations but extend them by showing that CONUT was independently associated with biopsy-based histologic activity and outperformed PNI in both UC and CD. Nonetheless, reported diagnostic performance of PNI and CONUT scores is heterogeneous across studies [12,52,53]. In this study, regression analyses showed that the CONUT score contributed more than PNI to the prediction of histologic activity. This difference is likely related to the inclusion of total cholesterol in the CONUT score, as pronounced lipid abnormalities were evident in our patients.

Chronic inflammation is well-known to alter lipid profiles: active IBD patients often exhibit lower HDL and total cholesterol levels compared to healthy controls [8]. In a cohort of IBD patients, complete mucosal healing was found to be positively associated with HDL levels [54]. However, we found no prior reports assessing the link between atherogenic lipid indices and disease activity. Beyond delivering an extensive parameter profile, this study delineates AIP as a predictive marker of histologic activity. Our results indicate that dysregulated lipid metabolism is associated with active bowel inflammation. Systemic inflammatory cytokines like TNF-α and IL-6 (abundant in active IBD) not only drive intestinal inflammation but also impair lipid metabolism, lowering HDL and raising triglycerides [55]. Conversely, pro-atherogenic lipoproteins can promote inflammation: oxidized LDL can activate macrophages and augment the inflammatory cascade [56]. Thus, metabolic imbalance and gut inflammation are bidirectionally linked. A previous study demonstrated a positive association between diet-induced inflammation and atherogenic indices [57]. It has also been reported that dietary lipids can trigger PPARδ and BCL6 expression in macrophages in the context of intestinal injury and LPS exposure, which in turn dampens IL-23 induction [58]. In colitis models, macrophages (fed by excess lipids) are among the first responders that amplify the inflammatory cascade [59]. Notably, AIP tends to increase with greater IBD activity (including more severe histologic inflammation), underscoring that ongoing mucosal inflammation fuels atherogenic lipid shifts [8]. Therefore, an elevated AIP in our patients likely signals an inflammatory, pro-atherogenic state that goes hand-in-hand with active IBD. This finding is clinically intriguing because it bridges gastroenterology with cardiometabolic health: IBD patients with active disease may benefit from addressing dyslipidemia not only to reduce cardiovascular risk but potentially to mitigate inflammation. In patients with IBD, AIP has been identified as an indicator of impaired coronary flow reserve and proposed as a useful tool for detecting those at risk of early atherosclerosis and coronary artery disease [13].

Although CONUT showed a higher AUC than AIP in ROC analyses, this does not make AIP redundant. Regression analysis demonstrated that both indices were independently associated with histologically active disease. These findings highlight the additional contribution of AIP. CONUT integrates serum albumin, lymphocyte count, and total cholesterol and therefore primarily reflects immunonutritional status [20]. By contrast, AIP captures the combined pattern of hypertriglyceridemia and reduced HDL cholesterol and is more closely related to lipoprotein particle size and plasma atherogenicity than to absolute cholesterol concentration alone [60,61]. In IBD, lipid disturbances extend beyond total cholesterol and include HDL- and triglyceride-related alterations associated with disease severity and mucosal healing [54,62]. Therefore, AIP may provide complementary information not fully represented by the cholesterol component of CONUT, even if CONUT demonstrated better overall discriminative performance in our cohort [13].

Notably, our analysis pinpointed three independent predictors of histologic activity, each representing a composite index of the above-discussed processes. These indices collectively capture the intricate links between inflammation (SIRI), nutritional status (CONUT score), and lipid metabolism (AIP) in IBD. These three indices underscore how malnutrition and dyslipidemia are not merely consequences of IBD but part of the disease activity spectrum. Poor nutritional status can intensify mucosal inflammation, while inflammation further worsens nutrition and perturbs lipid metabolism, perpetuating a detrimental cycle. The strong performance of our combined predictive model (with a high discriminative ability for active diseases) highlights the clinical utility of addressing these domains together. A high CONUT/low PNI may alert clinicians to a patient at risk of worse disease course or poor healing, prompting interventions such as nutritional support or more aggressive therapy. Second, SIRI (or similar indices like NLR or LMR) can serve as simple additions to disease activity monitoring. A rising SIRI might justify closer endoscopic evaluation or escalation of medical therapy before a patient flares severely. Third, the recognition of atherogenic dyslipidemia in active IBD suggests that patients should have periodic lipid profile checks. An elevated AIP not only indicates cardiovascular risk but, as our study suggests, mirrors intestinal inflammation. Clinicians might consider referring active IBD patients to a dietitian or preventive cardiology for lipid optimization, which could theoretically also dampen inflammatory drive. Overall, integrating these systemic biomarkers into clinical algorithms could improve the timing of therapy adjustments and potentially reduce reliance on invasive colonoscopies.

### Limitations

This study has several limitations. First, its retrospective design may have led to selection bias and limits the extent to which causal relationships can be inferred. Additionally, the retrospective design may have allowed the inclusion of patients with heterogeneous disease durations, which could affect the atherogenic profile independently of acute inflammatory processes. Second, the study was conducted at a single center. Third, the strict exclusion criteria, although applied to reduce non-IBD confounding of inflammatory, nutritional, and atherogenic indices, also yielded a selected cohort and therefore limit external validity. Excluding patients with cardiovascular/cerebrovascular disease, hepatic or renal failure, prior bowel surgery, and non-IBD immunosuppressive therapy may have underrepresented older, more comorbid, and metabolically more complex patients, potentially influencing the observed lipid and nutritional profiles. This issue is particularly relevant for atherogenic indices, which may be affected by baseline cardiovascular disease and related treatments. Fourth, because this was a retrospective study based on finalized pathology reports, detailed biopsy protocol information—such as the exact number, anatomical sites, and targeting of biopsy samples—was not consistently available in the records. Although UC histology was documented using the Nancy Index, CD histology was categorized as active disease versus remission according to predefined microscopic criteria rather than a formal validated histologic score. Finally, despite the wide systemic profiling in our study, regional and geographical differences may still shape IBD phenotypes and malnutrition risk by reflecting variability in the sociodemographic profiles of different populations [63,64,65]. Furthermore, the retrospective design precludes any firm conclusions about cause–effect pathways between nutritional, inflammatory, and atherogenic profiles. Future longitudinal studies in larger populations are warranted to elucidate these dynamics and to validate these results.

## 5. Conclusions

This study demonstrates that histologic disease activity in IBD is reflected by a distinct systemic profile encompassing inflammatory, nutritional, and atherogenic alterations. Out of the 44 analyzed variables, SIRI, the CONUT score, and AIP demonstrated the strongest associations with histologically active disease. These indices may facilitate the identification of subclinical disease activity and contribute to treatment decision-making. However, these findings should be interpreted cautiously due to the retrospective single-center design, strict exclusion criteria, and biopsy-related limitations. Prospective multicenter studies with larger cohorts are required to validate these findings and clarify their clinical utility.

## Figures and Tables

**Table 1:** Demographic and laboratory findings of study population.

Variables	All Population n = 100	CD	UC	*p*
		n = 50	n = 50	
Age, years	47.3 ± 13.7	46.8 ± 13.6	47.7 ± 13.9	0.733
Female gender, n (%)	57 (57.0)	29 (58.0)	28 (56.0)	0.840
BMI, kg/m^2^	26.1 ± 5.0	25.9 ± 4.5	26.2 ± 5.4	0.804
Smoking, n (%)	23 (23.0)	14 (28.0)	9 (18.0)	0.342
Disease activity scores	3.0 (1.0–6.0)	3.0 (1.0–5.0)	3.5 (1.0–6.0)	0.688
Histologic remission, n (%)				
Yes	40 (40.0)	21 (42.0)	19 (38.0)	0.683
No	60 (60.0)	29 (58.0)	31 (62.0)	
Localization for CD/UC, n (%)				
Ileal/Proctitis	–	22 (44.0)	24 (48.0)	–
Colonic/Left-sided colitis	–	19 (38.0)	15 (30.0)	
Ileocolonic/Extensive colitis	–	9 (18.0)	11 (22.0)	
Laboratory findings				
Leukocytes, ×10^3^ µL	8.1 ± 2.5	8.1 ± 2.6	8.1 ± 2.4	0.903
Neutrophils, ×10^3^ µL	4.7 (3.7–6.0)	4.9 (3.7–6.1)	4.6 (3.7–6.0)	0.548
Lymphocytes, ×10^3^ µL	1.6 (1.3–2.4)	1.6 (1.3–2.4)	1.7 (1.3–2.3)	0.717
Monocytes, ×10^3^ µL	0.6 ± 0.2	0.5 ± 0.2	0.6 ± 0.2	0.267
Eosinophils, ×10^3^ µL	0.1 (0.1–0.3)	0.1 (0.1–0.3)	0.1 (0.1–0.3)	0.682
Basophils, ×10^3^ µL	0.1 (0.0–0.1)	0.1 (0.0–0.1)	0.1 (0.0–0.1)	0.317
Hemoglobin, g/DL	12.4 ± 2.0	12.7 ± 1.8	12.1 ± 2.0	0.150
Hematocrit, %	37.6 ± 5.0	38.4 ± 5.0	36.8 ± 4.8	0.168
RDW, %	15.9 ± 3.2	16.3 ± 3.5	15.6 ± 2.9	0.263
RDWsd, fL	44.9 ± 8.8	46.0 ± 10.5	43.8 ± 6.6	0.200
Platelets, ×10^3^ µL	316.8 ± 85.1	310.1 ± 77.6	323.6 ± 84.2	0.478
MPV, fL	8.1 ± 0.8	8.0 ± 0.8	8.2 ± 0.8	0.294
Sedim, mm/h	20.5 (8.0–37.0)	21.0 (9.2–40.8)	18.0 (8.0–31.0)	0.506
CRP, mg/L	9.7 (4.3–26.3)	10.1 (4.2–29.1)	9.0 (4.5–22.6)	0.743
Albumin, g/dL	3.6 ± 1.0	3.5 ± 1.0	3.8 ± 1.0	0.140
Cholesterols, mg/dL	159.8 ± 46.5	153.8 ± 47.4	165.8 ± 45.3	0.201
HDL, mg/dL	44.5 ± 14.5	45.4 ± 14.2	43.5 ± 10.1	0.333
LDL, mg/dL	86.0 (66.5–112.0)	77.5 (65.5–128.2)	86.0 (67.2–122.0)	0.251
Triglycerides, mg/dL	107.5 (74.0–146.8)	117.0 (81.2–128.0)	99.0 (68.0–163.5)	0.694
ALT, IU/L	13.5 (9.0–18.0)	13.5 (8.0–17.0)	13.5 (10.0–19.0)	0.360
AST, IU/L	17.0 (12.0–22.0)	17.0 (12.0–22.0)	17.0 (12.0–20.8)	0.964
GGT, U/L	19.0 (13.0–29.2)	19.0 (14.0–29.0)	19.0 (13.0–29.8)	0.528
ALP, U/L	81.0 (67.0–93.0)	84.5 (67.0–93.0)	79.5 (70.2–86.0)	0.240
INR	1.0 ± 0.2	1.0 ± 0.1	1.1 ± 0.2	0.283
Urea	23.5 (19.0–29.0)	24.5 (19.0–29.0)	22.5 (17.2–32.2)	0.772
Creatinine, mg/dL	0.8 (0.6–0.9)	0.7 (0.6–0.9)	0.8 (0.6–0.9)	0.431
Total protein, g/L	7.0 ± 0.7	7.1 ± 0.8	6.9 ± 0.7	0.331
Inflammatory indices				
NLR	2.8 (1.9–4.4)	3.1 (1.8–5.2)	2.8 (2.1–4.2)	0.654
PLR	160.8 (131.0–238.6)	160.8 (133.9–226.9)	158.0 (133.3–238.6)	0.785
LMR	3.2 (2.2–4.5)	3.2 (2.7–4.5)	3.1 (2.0–4.4)	0.535
SII	784.0 (579.0–1364.8)	904.8 (549.0–1314.5)	759.4 (604.4–1331.0)	0.801
SIRI	1.4 (0.9–2.1)	1.4 (0.9–2.0)	1.6 (0.9–2.2)	0.577
AISI	430.1 (272.3–732.6)	423.9 (293.4–718.6)	470.4 (202.5–774.7)	0.915
CAR	2.3 (0.9–5.9)	2.1 (1.0–5.4)	2.5 (0.9–6.4)	0.893
Nutritional indices				
CONUT score	2.0 (1.0–4.0)	2.0 (1.0–4.0)	3.0 (2.0–4.0)	0.305
PNI	50.7 ± 8.3	50.4 ± 9.5	50.9 ± 7.1	0.792
Atherogenic lipid indices				
AIP	0.3 (0.2–0.6)	0.3 (0.2–0.6)	0.3 (0.2–0.6)	0.639
TG/HDL ratio	2.2 (1.5–3.7)	2.2 (1.7–3.6)	2.1 (1.4–4.0)	0.692

Data are mean ± standard deviation or median (IQR), or number (%). *p* < 0.05 indicates statistical significance. Abbreviations: AIP, atherogenic index of plasma; AISI, aggregate index of systemic inflammation; ALP, alkaline phosphatase; ALT, alanine aminotransferase; AST, aspartate aminotransferase; BMI, body mass index; CAR, C-reactive protein/albumin ratio; CD, Crohn’s disease; CONUT, controlling nutritional status; CRP, C-reactive protein; GGT, gamma-glutamyl transferase; HDL/LDL, high/low-density lipoprotein cholesterol; INR, international normalized ratio; IQR, interquartile range; LMR, lymphocyte-to-monocyte ratio; MPV, mean platelet volume; NLR, neutrophil-to-lymphocyte ratio; PNI, prognostic nutritional index; RDW/RDWsd, red cell distribution width/standard deviation; SII, systemic immune-inflammation index; SIRI, systemic inflammation response index; TG/HDL, triglyceride/HDL ratio; UC, ulcerative colitis.

**Table 2:** sPLS-DA model performance and top markers associated with histologic activity in Crohn’s disease and ulcerative colitis.

**Characteristics**	**Crohn’s Disease**			**Ulcerative Colitis**		
**% variation explained by latent factors**						
For predictor variables	0.80			0.83		
For outcome variables	0.72			0.74		
Num of used latent factors	1			1		
AUC (95% CI)	0.93 (0.83–1.00)			0.87 (0.76–0.99)		
Num of correctly classified (95% CI)	80% (70–94%)			83% (71–93%)		
*p*-value	<0.001			<0.001		
	**Factor**	**VIP**	**+/−**	**Factor**	**VIP**	**+/−**
**Top inflammatory markers** **responsible for outcome**	SIRI	1.71	+	SIRI	2.25	+
	LMR	1.68	−	CONUT score	2.20	+
	CONUT score	1.65	+	LMR	2.00	−
	AISI	1.49	+	AISI	2.08	+
	AIP	1.45	+	AIP	1.98	+
	PNI	1.39	−	PNI	1.58	−
	CAR	1.36	+	CAR	1.48	+
	LDL	1.14	+	LDL	1.32	+
	Hematocrit	1.11	−	Hematocrit	1.13	−
	Sedim	1.10	+	Sedim	1.10	+
	Hemoglobin	1.02	−	Hemoglobin	1.04	−

*p*-values are for the associations between outcome and latent factors. For predictor variables, “% variation explained” denotes the proportion of variance in systemic indices accounted for by the first sPLS-DA latent factor; for outcome variables, it denotes the proportion of variance in histologic activity explained. Model performance (AUC and proportion correctly classified) was evaluated using 5-fold stratified cross-validation, and 95% confidence intervals were obtained from 1000 bootstrap resamples of the cross-validated predictions. VIP, variable importance in projection; VIP ≥ 1.0 indicates variables with relevant contribution to the latent factor. “+/−” indicates positive or negative association with histologically active disease along the first sPLS-DA component. Abbreviations as in Table 1.

**Table 3:** Independent predictors for histologically active disease in IBD patients.

Variables	Crude Regression		Multivariable Regression	
	OR (95% CI)	*p*	OR (95% CI)	*p*
Crohn’s disease				
Hemoglobin	0.66 (0.45–0.96)	0.030 *	-	-
Hematocrit	0.85 (0.74–0.98)	0.011 *	-	-
Sedim	1.05 (1.01–1.09)	0.008 *	-	-
LDL	1.02 (1.01–1.04)	<0.001 *	-	-
LMR	0.38 (0.21–0.68)	<0.001 *	-	-
SIRI	1.23 (1.07–1.43)	<0.001 *	1.27 (1.07–1.49)	0.005 *
AISI	1.05 (1.01–1.09)	<0.001 *	-	-
CAR	1.22 (1.06–1.51)	<0.001 *	-	-
CONUT score	1.46 (1.01–2.05)	<0.001 *	1.57 (1.03–2.42)	0.018 *
PNI	0.92 (0.85–0.99)	<0.001 *	-	-
AIP	1.08 (1.03–1.13)	<0.001 *	1.05 (1.01–1.09)	0.023 *
			Nagelkerke R^2^ = 0.68	
Ulcerative colitis				
Hematocrit	0.88 (0.78–0.98)	<0.001 *	0.77 (0.62–0.96)	0.022 *
Hemoglobin	0.46 (0.28–0.78)	<0.001 *	-	-
Sedim	1.03 (1.01–1.05)	0.005 *	-	-
LDL	1.04 (1.01–1.06)	<0.001 *	-	-
LMR	0.61 (0.43–0.88)	<0.001 *	-	-
SIRI	1.31 (1.12–1.52)	<0.001 *	1.26 (1.06–1.50)	0.008 *
AISI	1.08 (1.03–1.14)	<0.001 *	-	-
CAR	1.29 (1.04–1.56)	<0.001 *	-	-
CONUT score	3.48 (1.58–7.71)	<0.001 *	4.21 (1.47–12.05)	0.006 *
PNI	0.90 (0.82–0.99)	<0.001 *	-	-
AIP	1.09 (1.04–1.14)	<0.001 *	1.09 (1.03–1.17)	0.006 *
			Nagelkerke R^2^ = 0.72	

Age, gender, BMI, smoking, and localization of illness were adjusted in all analyses. * *p* < 0.05 indicates statistical significance. Abbreviations: AIP, atherogenic index of plasma; AISI, aggregate index of systemic inflammation; CAR, C-reactive protein-to-albumin ratio; CI, confidence interval; CONUT, controlling nutritional status; LDL, low-density lipoprotein cholesterol; LMR, lymphocyte-to-monocyte ratio; OR, odds ratio; PNI, prognostic nutritional index; Sedim, erythrocyte sedimentation rate; SIRI, systemic inflammation response index.

**Table 4:** Diagnostic performance of independent variables for predicting histologically active disease in IBD patients.

Variables	Cutoff	Sens. (%)	Spec. (%)	PPV	NPV	AUC (95% CI)
CD						
CONUT score	>4.0	79.1	70.2	79.3	71.4	0.78 (0.66–0.91)
SIRI	>1.1	87.0	67.9	78.1	77.8	0.86 (0.75–0.97)
AIP	>0.2	60.2	82.8	81.0	58.6	0.73 (0.59–0.86)
UC						
CONUT score	>4.0	71.8	79.0	84.6	62.5	0.72 (0.62–0.83)
SIRI	>1.2	72.5	87.8	91.7	65.4	0.78 (0.64–0.92)
AIP	>0.3	51.0	78.3	80.0	50.0	0.60 (0.52–0.69)

Abbreviations: AIP: atherogenic index of plasma; AUC: area under the curve; CD: Crohn’s disease; CI: confidence interval; CONUT: controlling nutritional status; NPV: negative predictive value; PPV: positive predictive value; Sens.: sensitivity; Spec.: specificity; SIRI: systemic inflammation response index; UC: ulcerative colitis.

## Data Availability

The data that support the findings of this study are available on request from the corresponding author.

## Supplementary Materials

The following supporting information can be downloaded at https://www.mdpi.com/article/10.3390/biomedicines14040740/s1, Table S1: Inflammatory, nutritional, and atherogenic blood-based markers assessed in the study. Table S2: Formulas of composite inflammatory, nutritional, and atherogenic indices. Table S3: Demographic and clinical characteristics of Crohn’s disease patients with histologic remission and active disease. Table S4: Demographic and clinical characteristics of ulcerative colitis patients with histologic remission and active disease.
