# Supplementary material for: Inflammatory, Nutritional, and Atherogenic Profiles Associated with Histologic Activity in Inflammatory Bowel Disease
Source: Biomedicines. 2026 Mar 24;14(4):740. doi: 10.3390/biomedicines14040740 (PMC13113708; doi:10.3390/biomedicines14040740)
Supplement: Supplementary file 1 [file biomedicines-14-00740-s001.zip › biomedicines-4162935-Supplementary Tables.pdf]

Supplementary Table S1. Inflammatory, nutritional, and atherogenic blood-based markers assessed in the study.

Class	Variables
**Demographic**	Age, gender, BMI, smoking
**IBD phenotype**	Disease type (Ulcerative colitis vs Crohn's disease), UC extent (proctitis, left-sided colitis, extensive colitis), CD location (ileal, colonic, ileocolonic)
**Systemic inflammatory cell counts**	Leukocytes, Neutrophils, Lymphocytes, Monocytes, Eosinophils, Basophils, Platelets
**Red blood cell and platelet indices**	Hemoglobin, Hematocrit, Red cell distribution width (RDW, RDW-SD), Mean platelet volume (MPV)
**Acute-phase and biochemical**	C-reactive protein (CRP), Erythrocyte sedimentation rate (Sedim), Alanine aminotransferase (ALT), Aspartate aminotransferase (AST), Gamma-glutamyl transferase (GGT), Alkaline phosphatase (ALP), International normalized ratio (INR), Urea, Creatinine, Total protein, Albumin
**CBC-derived inflammatory indices**	Neutrophil-to-lymphocyte ratio (NLR),<br>Platelet-to-lymphocyte ratio (PLR),<br>Lymphocyte-to-monocyte ratio (LMR),
**Composite inflammatory indices**	Systemic immune-inflammation index (SII)<br>Systemic inflammation response index (SIRI),<br>Aggregate index of systemic inflammation (AISI)
**CRP–albumin—based indices**	CRP-to-albumin ratio (CAR)
**Nutritional indices**	Prognostic nutritional index (PNI),<br>Controlling Nutritional Status (CONUT) score
**Lipid profile**	Total cholesterol,<br>High-density lipoprotein (HDL) cholesterol,<br>Low-density lipoprotein (LDL) cholesterol,<br>Triglycerides
**Atherogenic lipid indices**	Atherogenic index of plasma (AIP),<br>Triglyceride-to-HDL ratio (TG/HDL)

All parameters were calculated from fasting blood samples obtained on the same day as endoscopy and biopsy.

Supplementary Table S2. Formulas of composite inflammatory, nutritional, and atherogenic indices.

Index	Formula
**NLR**	Neutrophils / Lymphocytes
**PLR**	Platelets / Lymphocytes
**LMR**	Lymphocytes / Monocytes
**SII**	Platelets × Neutrophils) / Lymphocytes
**SIRI**	(Neutrophils × Monocytes) / Lymphocytes
**AISI**	(Neutrophils × Monocytes × Platelets) / Lymphocytes
**CAR**	CRP / Albumin
**PNI**	10 × Albumin + 5 × Lymphocytes
**CONUT score**	Albumin score + Lymphocytes score + cholesterol score
**AIP**	$\log_{10}$(Triglycerides / HDL)
**TG/HDL**	Triglycerides / HDL

Supplementary Table S3. Demographic and clinical characteristics of Crohn's disease patients with histologic remission and active disease.

Variables	Crohn's Disease		p
	Histologic remission n=21	Active disease n=29	
Age, years	49.7 ± 13.1	44.7 ± 13.7	0.199
Female gender, n (%)	11 (52.4)	18 (62.1)	0.493
BMI, kg/m^2	24.8 ± 4.8	26.7 ± 4.2	0.139
Smoking, n (%)	6 (28.6)	8 (27.6)	0.939
Disease activity score	3.0 (1.0 – 4.0)	4.5 (3.0 – 6.0)	<0.039*
Localization, n (%)			
Ileal	10 (47.6)	12 (41.4)	0.825
Colonic	8 (38.1)	11 (37.9)	
Ileocolonic	3 (14.3)	6 (20.7)	
Laboratory findings			
Leukocytes, × 10^3 μL	6.9 ± 1.9	8.9 ± 2.8	0.008*
Neutrophils, × 10^3 μL	4.5 (2.8 – 4.7)	5.7 (4.6 – 7.5)	<0.001*
Lymphocytes, × 10^3 μL	2.1 (1.3 – 2.6)	1.5 (1.1 – 2.2)	0.004*
Monocytes, × 10^3 μL	0.5 ± 0.1	0.7 ± 0.3	0.003*
Eosinophils, × 10^3 μL	0.1 (0.1 – 0.3)	0.1 (0.1 – 0.2)	0.427
Basophils, × 10^3 μL	0.1 (0.0 – 0.1)	0.1 (0.0 – 0.1)	0.299
Hemoglobin, g/DL	13.4 ± 1.4	12.2 ± 1.9	0.023*
Hematocrit, %	40.4 ± 3.5	37.0 ± 5.5	0.011*
RDW, %	16.5 ± 4.9	16.2 ± 2.3	0.760
RDWsd, fL	46.9 ± 12.4	45.4 ± 9.2	0.639
Platelets, × 10^3 μL	301.3 ± 59.5	316.4 ± 89.0	0.502
MPV, fL	8.2 ± 0.5	7.9 ± 0.9	0.145
Sedim, mm/h	11.0 (3.0 – 21.0)	33.0 (17.0 – 42.0)	0.003*
CRP, mg/L	6.3 (3.6 – 11.5)	19.9 (5.8 – 70.3)	0.007*
Albumin, g/dL	4.3 ± 0.3	2.9 ± 0.8	<0.001*
Cholesterols, mg/dL	135.1 ± 48.7	160.4 ± 45.8	0.040*
HDL, mg/dL	47.1 ± 14.8	40.3 ± 15.0	0.031*
LDL, mg/dL	71.0 (65.0 – 104.0)	91.0 (87.0 – 123.0)	<0.001*
Triglycerides, mg/dL	82.0 (74.0 – 117.0)	123.0 (100.0 – 140.0)	<0.001*
ALT, IU/L	15.0 (12.0 – 15.0)	16.0 (8.0 – 18.0)	0.231
AST, IU/L	17.0 (16.0 – 26.0)	16.0 (11.0 – 24.0)	0.247
GGT, U/L	19.0 (16.0 – 30.0)	19.0 (13.0 – 29.0)	0.768
ALP, U/L	84.0 (80.0 – 90.0)	86.0 (66.0 – 99.0)	0.875
INR	1.0 ± 0.1	1.0 ± 0.1	0.318
Urea	28.0 (19.0 – 29.0)	24.0 (19.0 – 28.0)	0.686
Creatinine, mg/dL	0.7 (0.7 – 0.9)	0.8 (0.6 – 0.9)	0.624
Total protein, g/L	7.3 ± 0.6	6.9 ± 0.9	0.157
Inflammatory indices			
NLR	1.8 (1.5 – 3.2)	4.0 (2.7 – 6.8)	<0.001*
PLR	160.8 (128.8 – 207.9)	193.8 (145.6 – 290.0)	0.120
LMR	4.5 (3.2 – 6.0)	2.7 (1.7 – 3.9)	<0.001*
SII	579.0 (489.0 – 956.1)	1104.9 (695.4 – 2296.0)	<0.001*
SIRI	0.9 (0.6 – 1.4)	1.8 (1.2 – 3.0)	<0.001*
AISI	293.4 (131.1 – 423.9)	488.8 (217.2 – 639.6)	<0.001*
CAR	1.5 (0.8 – 2.6)	3.0 (1.3 – 5.8)	<0.001*
Nutritional indices			
CONUT score	1.0 (0.0 – 2.0)	2.0 (1.0 – 4.0)	<0.001*
PNI	54.0 ± 7.1	47.9 ± 10.3	<0.001*
Atherogenic lipid indices			
AIP	0.2 (0.1 – 0.3)	0.5 (0.4 – 0.6)	<0.001*
TG/HDL ratio	1.5 (1.4 – 1.9)	3.0 (2.3 – 3.9)	<0.001*

Data are mean ± standard deviation or median (IQR), or number (%). *$p<0.05$ indicates statistical significance. Abbreviations: AIP, atherogenic index of plasma; AISI, aggregate index of systemic inflammation; ALP, alkaline phosphatase; ALT, alanine aminotransferase; AST, aspartate aminotransferase; BMI, body mass index; CAR, C-reactive protein/albumin ratio; CD, Crohn's disease; CONUT, Controlling Nutritional Status; CRP, C-reactive protein; GGT, gamma-glutamyl transferase; HDL/LDL, high/low-density lipoprotein cholesterol; INR, international normalized ratio; IQR, interquartile range; LMR, lymphocyte-to-monocyte ratio; MPV, mean platelet volume; NLR, neutrophil-to-lymphocyte ratio; PNI, Prognostic Nutritional Index; RDW/RDWsd, red cell distribution width/standard deviation; SII, systemic immune-inflammation index; SIRI, systemic inflammation response index; TG/HDL, triglyceride/HDL ratio; UC, ulcerative colitis.

Supplementary Table S4. Demographic and clinical characteristics of ulcerative colitis patients with histologic remission and active disease.

Variables	Ulcerative Colitis		p
	Histologic remission n = 19	Active disease n = 31	
Age, years	51.2 ± 13.3	48.5 ± 13.8	0.499
Female gender, n (%)	10 (52.6)	18 (58.1)	0.707
BMI, kg/m²	27.0 ± 5.2	25.7 ± 5.6	0.413
Smoking, n (%)	3 (15.8)	6 (19.4)	0.750
Disease activity score	2.5 (1.0 – 4.0)	5.0 (3.0 – 6.0)	0.024*
Localization, n (%)			
Proctitis	10 (52.6)	14 (45.2)	
Left-sides colitis	6 (31.6)	9 (29.0)	0.705
Extensive colitis	3 (15.8)	8 (25.8)	
Laboratory findings			
Leukocytes, × 10³ μL	7.2 ± 2.5	8.7 ± 2.4	0.039*
Neutrophils, × 10³ μL	4.1 (3.0 – 4.4)	5.3 (4.2 – 6.3)	0.007*
Lymphocytes, × 10³ μL	1.9 (1.4 – 2.3)	1.5 (1.1 – 2.0)	0.023*
Monocytes, × 10³ μL	0.5 ± 0.2	0.7 ± 0.2	<0.001*
Eosinophils, × 10³ μL	0.1 (0.1 – 0.3)	0.1 (0.1 – 0.3)	0.827
Basophils, × 10³ μL	0.1 (0.0 – 0.1)	0.1 (0.0 – 0.1)	0.595
Hemoglobin, g/DL	13.4 ± 1.2	11.4 ± 2.1	<0.001*
Hematocrit, %	38.8 ± 4.5	34.7 ± 4.8	<0.001*
RDW, %	14.7 ± 2.2	16.1 ± 3.1	0.101
RDWsd, fL	44.2 ± 4.4	43.5 ± 7.7	0.705
Platelets, × 10³ μL	307.8 ± 95.1	333.3 ± 93.4	0.433
MPV, fL	8.2 ± 0.9	8.3 ± 0.8	0.721
Sedim, mm/h	12.0 (7.5 – 20.5)	24.0 (10.5 – 33.0)	0.004*
CRP, mg/L	4.9 (3.6 – 11.5)	14.7 (4.5 – 26.5)	0.008*
Albumin, g/dL	4.4 ± 0.2	3.4 ± 1.0	<0.001*
Cholesterols, mg/dL	145.3 ± 44.5	174.6 ± 42.2	0.039*
HDL, mg/dL	46.6 ± 10.3	40.1 ± 10.1	0.033*
LDL, mg/dL	74.0 (62.0 – 95.5)	98.0 (74.0 – 119.5)	<0.001*
Triglycerides, mg/dL	68.0 (51.0 – 82.5)	146.0 (99.0 – 200.0)	<0.001*
ALT, IU/L	12.0 (8.5 – 17.0)	14.0 (10.0 – 21.5)	0.340
AST, IU/L	17.0 (12.0 – 19.5)	17.0 (12.5 – 22.0)	0.400
GGT, U/L	19.0 (13.0 – 25.5)	19.0 (13.0 – 37.5)	0.681
ALP, U/L	77.0 (66.0 – 85.5)	80.0 (70.5 – 98.0)	0.624
INR	1.0 ± 0.1	1.1 ± 0.2	0.723
Urea	25.0 (20.0 – 33.0)	21.0 (16.5 – 29.5)	0.298
Creatinine, mg/dL	0.8 (0.6 – 1.1)	0.8 (0.7 – 0.9)	0.887
Total protein, g/L	7.1 ± 0.5	6.8 ± 0.8	0.127
Inflammatory indices			
NLR	2.4 (1.8 – 3.0)	2.9 (2.2 – 4.5)	0.043*
PLR	153.2 (141.6 – 231.7)	161.5 (125.5 – 262.1)	0.711
LMR	4.0 (2.6 – 6.8)	2.5 (1.9 – 3.3)	<0.001*
SII	668.0 (525.1 – 784.0)	834.0 (659.6 – 1560.9)	0.040*
SIRI	0.9 (0.5 – 1.4)	2.0 (1.5 – 3.0)	<0.001*
AISI	202.5 (150.3 – 369.6)	581.0 (417.5 – 1092.7)	<0.001*
CAR	1.1 (0.8 – 2.7)	2.1 (1.0 – 8.0)	<0.001*
Nutritional indices			
CONUT score	2.0 (1.5 – 2.0)	3.0 (2.0 – 4.0)	<0.001*
PNI	53.6 ± 4.8	49.2 ± 7.8	0.034*
Atherogenic lipid indices			
AIP	0.2 (0.1 – 0.3)	0.5 (0.3 – 0.7)	<0.001*
TG/HDL ratio	1.4 (1.2 – 2.0)	3.4 (2.0 – 4.7)	<0.001*

Data are mean ± standard deviation or median (IQR), or number (%). *$p<0.05$ indicates statistical significance. Abbreviations: AIP, atherogenic index of plasma; AISI, aggregate index of systemic inflammation; ALP, alkaline phosphatase; ALT, alanine aminotransferase; AST, aspartate aminotransferase; BMI, body mass index; CAR, C-reactive protein/albumin ratio; CD, Crohn's disease; CONUT, Controlling Nutritional Status; CRP, C-reactive protein; GGT, gamma-glutamyl transferase; HDL/LDL, high/low-density lipoprotein cholesterol; INR, international normalized ratio; IQR, interquartile range; LMR, lymphocyte-to-monocyte ratio; MPV, mean platelet volume; NLR, neutrophil-to-lymphocyte ratio; PNI, Prognostic Nutritional Index; RDW/RDWsd, red cell distribution width/standard deviation; SII, systemic immune-inflammation index; SIRI, systemic inflammation response index; TG/HDL, triglyceride/HDL ratio; UC, ulcerative colitis.
